# Less Virulent *Leptosphaeria biglobosa* Immunizes the Canola Plant to Resist Highly Virulent *L. maculans,* the Blackleg Pathogen

**DOI:** 10.3390/plants11070996

**Published:** 2022-04-06

**Authors:** Kaluhannadige Rasanie Eranka Padmathilake, Wannakuwattewaduge Gerard Dilantha Fernando

**Affiliations:** Department of Plant Science, University of Manitoba, Winnipeg, MB R3T 2N2, Canada; padmatkr@myumanitoba.ca

**Keywords:** *Brassica napus*, *Leptosphaeria maculans*, *Leptosphaeria biglobosa*, biological control

## Abstract

*Leptosphaeria biglobosa* is a less virulent *Leptosphaeria* spp. that causes blackleg disease in canola. Previous studies from our lab have shown that inoculation with the less virulent *L. biglobosa* can boost the resistance of canola plants against the highly virulent *L. maculans*. The objective of this study was to confirm the effectiveness of *L. biglobosa* as a biocontrol agent against *L. maculans* utilizing morphology, fluorescence microscopy, gene quantification, and transcriptomic analysis. The in planta development of two *Leptosphaeria* species inoculated at different time points was assessed using fluorescent protein-tagged isolates which are GFP-tagged *L. maculans* and DsRed-tagged *L. biglobosa*. The growth inhibition of *L. maculans* by pre-and co-inoculated *L. biglobosa* was supported by no lesion development on cotyledons and no or weak fluorescence protein-tagged mycelia under the confocal microscope. The host defense-related genes, *WRKY33*, *PR1*, *APX6*, and *CHI,* were upregulated in *L. biglobosa* inoculated Westar cotyledons compared to *L. maculans* inoculated cotyledons. The quantification of each pathogen through qPCR assay and gene expressions analysis on host defense-related genes by RT-qPCR confirmed the potential of *L. biglobosa* “brassicae’ in the management of the blackleg disease pathogen, *L. maculans* ‘brassicae’, in canola.

## 1. Introduction

Blackleg is one of the most destructive diseases in canola (*Brassica napus* L.) growing areas and causes more than CAD $900 million annual loss per growing season worldwide [1]. Two *Leptosphaeria* species are associated with blackleg [2], and they are *Leptosphaeria maculans. L. maculans* isolates have been categorized into two sub clades ‘brassicae’ and ‘lepidii’, while *L. biglobosa* have been categorized into seven isolate groups, and they are ‘brassicae’, ‘canadensis’, ‘thlaspii’, ‘erysimii’, ‘australensis’, ‘occiaustralensis’, and ‘americensis’, respectively [2,3,4]. *L. biglobosa* ‘brassicae’ is the most common species and has been found in most canola growing regions [5]. These species co-exist in most *B. napus* growing areas [6].

*L. maculans* and *L. biglobosa* were described as virulent and less virulent strains, respectively [7]. Although *L. maculans* is more aggressive than *L. biglobosa*, their life cycles are similar [8]. The two species are genetically different and exhibit distinct disease symptoms on the canola plant [7]. *L*. *maculans* produces gray lesions with dark brown margins and black pycnidia. The pathogen moves towards the stem through the leaf petiole to the stem base and causes the main characteristic symptom of the disease, a blackened stem base canker, which was the reason for the name of the disease, ‘blackleg’. *L. biglobosa* caused leaf lesions to be smaller, darker, and confined to the upper stem [9]. *L. maculans* is a hemibiotrophic fungus, while *L. biglobosa* is a necrotrophic fungus with a comparatively shorter biotrophic stage in its life cycle. Furthermore, *L. biglobosa* is known to start its expression of hydrolases, a characteristic for the necrotrophic stage earlier than *L. maculans* [10].

Several studies demonstrated the resistance induction of the canola plant by *L. biglobosa* and suppression of *L. maculans* infection when *L. biglobosa* was pre- or co-inoculated with *L. maculans*. This suppression may be due to (1) the competition arising between the two species during infection and colonization within the host plant and (2) the induction of resistance in the host plant [11,12]. The enhanced plant defense in the host activated by biotic or abiotic factors is induced resistance [13]. Induction of disease resistance by pre- or co-inoculation with an avirulent or less virulent strain has been shown against many pathogenic strains and is known to be a broad-spectrum defense that protects the host from subsequent infections. For instance, the hampering of control strategies against the virulent *Fusarium graminearum* in the disease complex causes FHB by the presence of the less pathogenic *F. poae* [14] and tomato biocontrol activity of the *F. oxysporum* Fo47 endophyte against the pathogen *F. oxysporum* f. sp. *Lycopersici* causes tomato wilt disease [15]. The activated defense responses include (1) oxidative burst, which can cause cell death by restricting the pathogen at the inoculation site [16], and (2) changes in the cell wall composition by inhibiting pathogen penetration and secondary metabolite production, which can act as antimicrobial compounds [17].

Studying defense-related gene expressions is expected in the investigation of different plant–pathogen interactions. Phytohormones, such as salicylic acid (SA), ethylene (ET), and jasmonic acid (JA), coordinate defense responses by the activation of defense-related gene expressions. SA-mediated defense response plays a central role in local and systemic acquired resistance (SAR) against biotrophic pathogens. The ET/JA-mediated response contributes to the defense against necrotrophic pathogens. SA and JA target genes are *PR1* and *PDF1.2*, respectively [18,19]. The respiratory burst oxidase homologues (RBOHs) are a major source of reactive oxygen species (ROS) during plant–microbe interactions [20]. On the other hand, ascorbate peroxidases (APXs) are another critical group of enzymes adjusting ROS levels in the cells [21]. Furthermore, transcription factors play important roles in defense responses. Transcription factor WRKY33 is essential for defense toward the necrotrophic pathogens [22], and plant-specific transcription factor WRKY70 is a common component in SA- and JA-mediated signal pathways [18].

The focus of this study was to investigate the potential of *L. biglobosa* in inducing canola plant resistance against the virulent species *L. maculans* through a combination of (1) the assessment of outer lesion developments, (2) confocal microscopic observations of fluorescent protein-tagged isolate growth in planta, (3) the quantification of each isolate by quantitative PCR, and (4) defense-related host gene expressions RT-qPCR.

## 2. Results

### 2.1. Disease Development in Cotyledons

**Figure 1:**
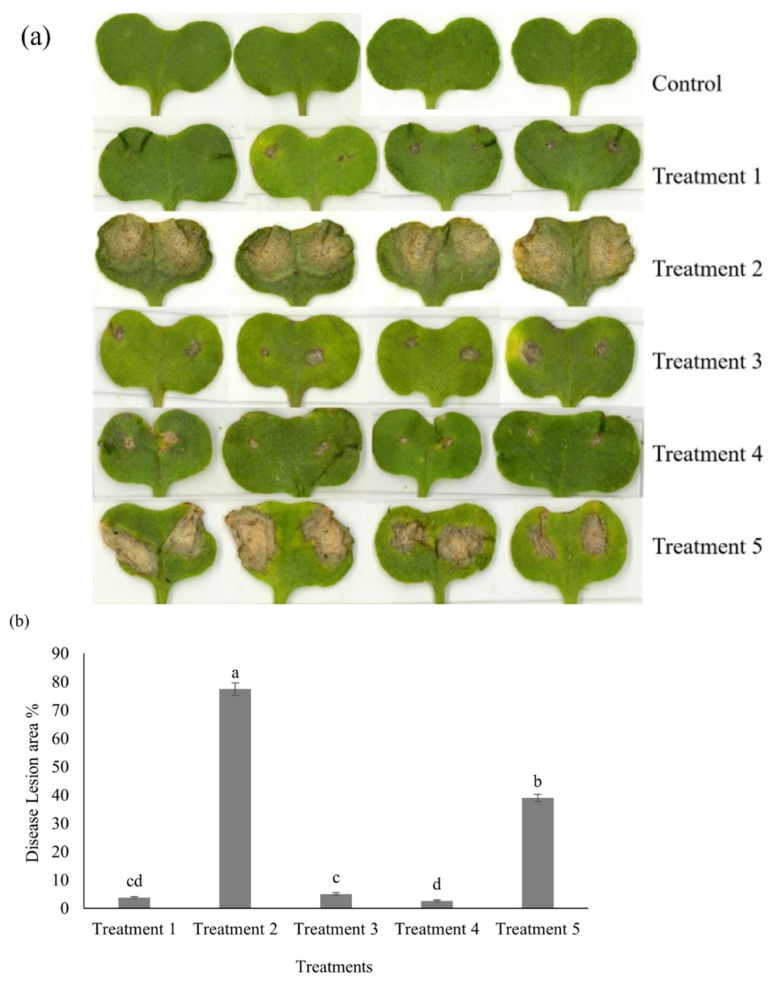
Blackleg disease lesion development in Westar cotyledons at 14 days post-inoculation under controlled conditions with *Leptosphaeria maculans* ‘brassicae’ and *L. biglobosa* ‘brassicae’ inoculations. Seven-day-old cotyledons were wound inoculated and observed the lesion development in time course. Cotyledons inoculated with only GFP-tagged *L. maculans* (LmGFP) and cotyledons pre-inoculated with LmGFP showed significantly higher lesion development at 14 dpi. Control: distilled water treatment; Treatment 1: LbDsRed inoculation; Treatment 2: LmGFP inoculation; Treatment 3: LbDsRed and LmGFP co-inoculation; Treatment 4: LbDsRed followed by LmGFP inoculation (in 24 h intervals); Treatment 5: LmGFP followed by LbDsRed inoculation (in 24 h intervals). (**a**) Scanned images of disease lesion development in cotyledons; (**b**) Lesion size shown as a percentage of the total leaf area of both host types calculated by ImageJ. Different letters refer to significant differences between treatments (*p* ≤ 0.05).

The disease lesion caused by LbDsRed was limited to the surrounding inoculation sites of each lobe of the cotyledon (Figure 1). The significantly largest and second-largest disease lesions were observed in cotyledons in which *L. maculans* was used as the sole inoculum and pre-inoculum, as shown in Figure 1. In those two treatments, black colored pycnidia were visible on the disease lesions. Lesion development on LbDsRed and LmGFP co-inoculated cotyledons and LbDsRed pre-inoculated cotyledons were not statistically different from the lesions seen in LmDsRed solely inoculated cotyledons (*p* < 0.05) (Figure 1).

### 2.2. Presence of Leptosphaeria spp. in Inoculated Westar Cotyledons

**Figure 2:**
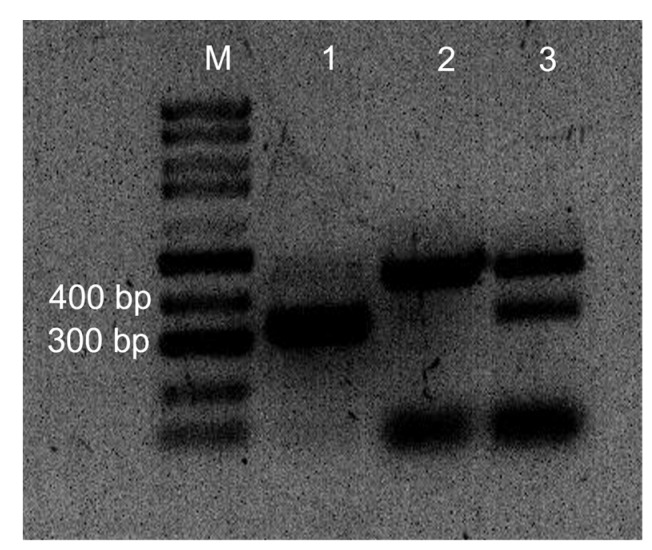
Gel image of species-specific PCR products of *Leptosphaeria maculans* ‘brassicae’ and *L. biglobosa* ‘brassicae’ isolates in inoculated Westar cotyledons. PCR identification of *L. biglobosa* and *L. maculans* with species-specific primers, LmacF/LmacR and LbigF/LmacR in a multiplex PCR. *L. biglobosa* ‘brassicae’ and *L. maculans* ‘brassicae’ gave PCR bands of 444 bp and 331 bp, respectively. PCR products were resolved by electrophoresis through a 1% (*w*/*v*) agarose gel. Lane 1: *L. maculans* ‘brassicae’ inoculated cotyledon gDNA; Lane 2: *L. biglobosa* ‘brassicae’ inoculated cotyledon gDNA; Lane 3: both isolates co-inoculated cotyledon gDNA; M: 100 bp DNA ladder.

PCR products of genomic DNA extracted from infected cotyledons inoculated with only LmGFP and only LbDsRed showed gel bands of 444 bp and 331 bp sizes for LbDsRed and LmGFP, respectively. Co-inoculated cotyledons with both isolates displayed both bands (Figure 2).

### 2.3. L. biglobosa Inhibits the L. maculans Growth in B. napus Cotyledons

**Figure 3:**
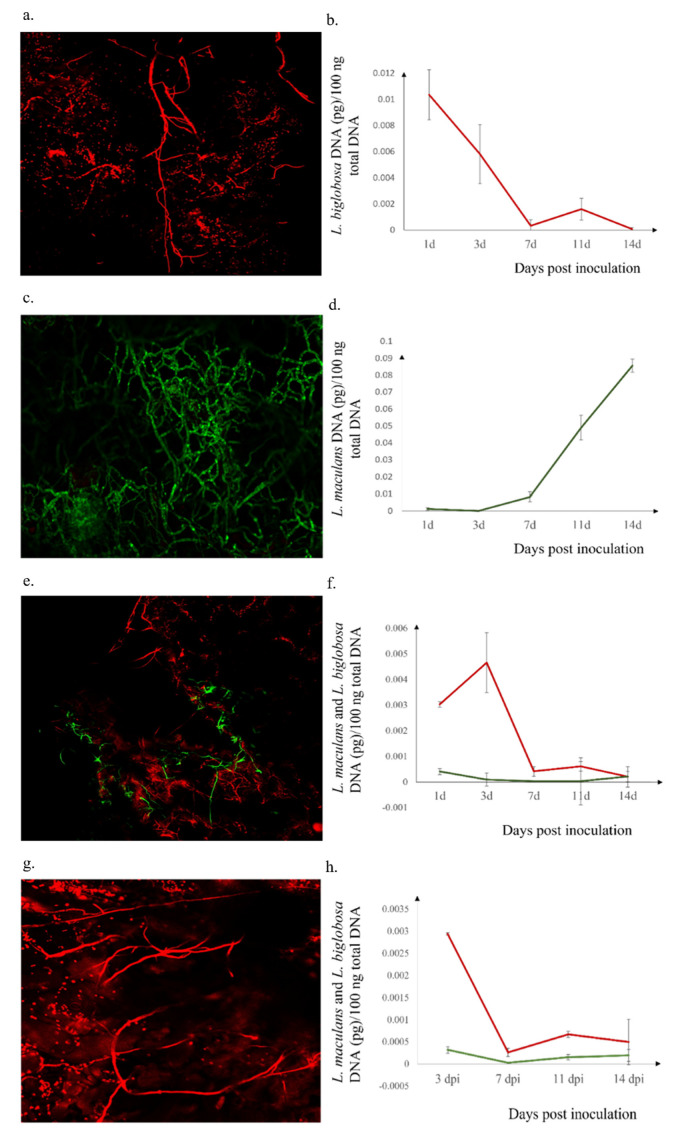

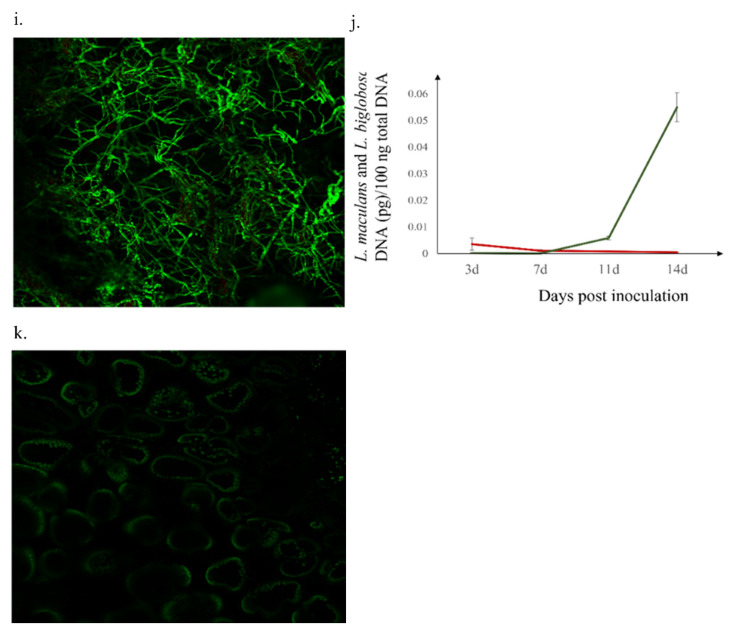
DsRed-tagged *L. biglobosa* (LbDsRed) and GFP-tagged *L. maculans* (LmGFP) in planta development of Westar cotyledons under controlled environment at 14 days post-inoculation. (**a**) Confocal microscopic observations of fluorescence protein-tagged *Leptosphaeria* isolates (10× magnification). (**b**) Quantification of each isolate in planta at 3-, 7-, 11-, and 14- dpi. LbDsRed and LmGFP mycelia shown in fluorescent red and green colors, respectively. In graphs, red and green colors represent LbDsRed and LmGFP DNA, respectively. (**a**,**b**) LbDsRed inoculation; (**c**,**d**) LmGFP inoculation; (**e**,**f**) LbDsRed and LmGFP co-inoculation; (**g**,**h**) LbDsRed followed by LmGFP inoculation with one day interval; (**i**,**j**) LmGFP followed by LbDsRed inoculation with one-day interval; (**k**) Chlorophyll autofluorescence of cellular chloroplasts of cotyledons in control.

Confocal observation at 14 dpi showed the LbDsRed mycelia, but, comparatively, in less density (Figure 3a). The DNA quantity of LbDsRed was reduced approximately 33 times from 1- to 7- dpi (Figure 3b). In cotyledons inoculated solely by LmGFP, confocal microscopic observations showed a densely grown mass of LmGFP (Figure 3c). The *L. maculans* DNA quantity increased starting from three dpi onwards. Moreover, the fungal DNA quantity was raised at an exponential rate from seven dpi onwards (Figure 3d). LmGFP DNA increased 10 times from 7- to 14- dpi. The co-inoculated cotyledons displayed both LbDsRed and LmGFP signals, as shown in Figure 3e. The quantification study demonstrated the reduction in LbDsRed in a similar pattern in cotyledons treated only with LbDsRed. However, the reduction in the DNA of LmGFP was also observed in those cotyledons (Figure 3f).

Cotyledons pre-inoculated with LbDsRed one day before the LmGFP inoculation showed only LbDsRed mycelia in confocal observations. The quantification study demonstrated clear growth inhibition of LmGFP from 3- to 14- dpi (Figure 3h). On the other hand, LmGFP inoculation followed with LbDsRed inoculation with a 1-day interval showed densely developed LmGFP in confocal images and increment in LmGFP DNA quantity starting from 7 dpi similar to the LmGFP solely treated cotyledons. LbDsRed was not observed in the confocal observations, and the DNA quantity of LbDsRed reduced with time (Figure 3j).

### 2.4. Expression of Defense Genes

**Figure 4:**
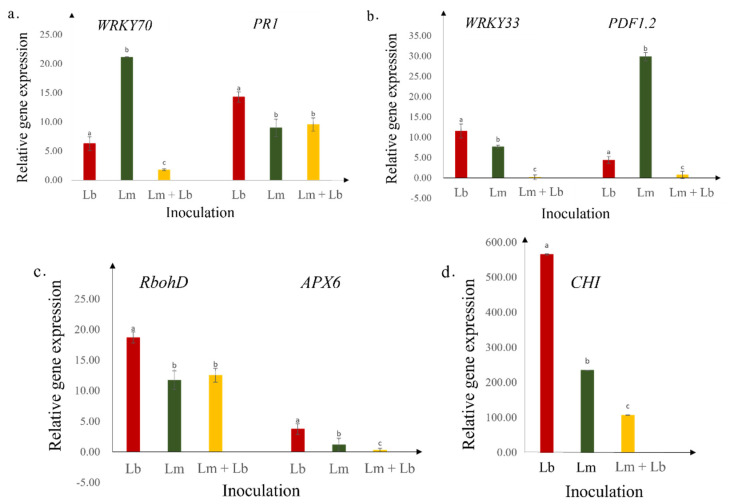
Relative transcript levels of several important genes involved in host defense signaling pathways were assessed by quantitative RT-PCR at 7 days post-inoculation. The expression of *WRKY70* and *PDF1.2* showed significantly higher expressions in Lm solely inoculated cotyledons. *PR1*, *WRKY33*, *RbohD*, *APX6*, and *CHI* showed significantly higher expression in cotyledons inoculated with only Lb. (**a**) Relative gene expression of *WRKY70* and *PR1*; (**b**) Relative gene expression of *WRKY33* and *PDF1.2*; (**c**) Relative gene expression of *RbohD* and *APX6*; (**d**) Relative gene expression of *CHI*; in Westar cotyledons inoculated with only DsRed-tagged *L. biglobosa* (Lb), with only GFP-tagged *L. maculans* (Lm) and co-inoculated with both isolates (Lm + Lb). Different letters refer to significant differences (*p* ≤ 0.05).

**Figure 5:**
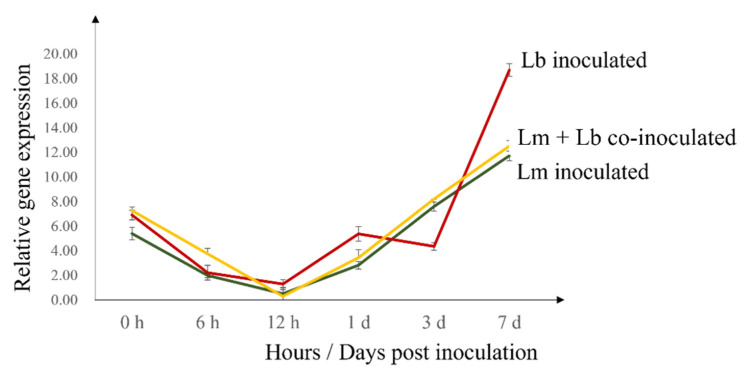
Expression profile of *RbohD* in Westar cotyledons infected with LmGFP, LbDsRed as sole and combined inocula. *RbohD* expression was upregulated at an exponential rate from 3- to 7- dpi. The observations were taken at 0-, 6-, 12- hpi and 1-, 3-, 7- dpi.

Except for *WRKY70* and *PDF1.2*, all other genes, *PR1, WRKY33, RbohD, APX6,* were upregulated in *L. biglobosa* inoculated cotyledons compared to the other two methods of inoculations. *CHI,* chitinase, which is important in the degradation of pathogen cell walls, was expressed significantly higher in *L. biglobosa* inoculated cotyledons (Figure 4). As shown by Figure 5, the ROS burst marker gene, *RbohD,* started to upregulate starting from 3 dpi onwards at an exponential rate.

## 3. Discussion

This study demonstrated the resistance inducing potential of a less virulent isolate, *L. biglobosa* ‘brassicae’ in the susceptible canola variety Westar against virulent *L. maculans* ‘brassicae’. The successful control of *L. maculans* was demonstrated by pre-and co-inoculation of *L. biglobosa* by studies of cotyledon lesion development, in planta mycelial development, pathogen quantification, and gene expressions related to host defense.

The avirulence nature of *L. biglobosa* ‘brassicae’ on the susceptible Westar was evidenced by (1) the restricted disease lesion at the inoculation site, (2) reduction of LbDsRed DNA quantity by 33 folds from 3- to 7- dpi, and (3) low density of *L. biglobosa* mycelia under the confocal microscope. The restricted disease lesion caused by *L. biglobosa* could be related to the oxidative burst that arises in the host plant during the pathogen infection [10]. Lowe et al., (2014) demonstrated that the oxidative burst occurrence is higher in the host infected with *L. biglobosa* than in the host infected by *L. maculans*. *RbohD* is the oxidative burst marker gene of canola and RbohD-dependent ROS is involved in the plant defense [23]. *RbohD* was upregulated starting from 3 dpi onwards in this study. As the principal ROS producer, *RbohD* leads the hypersensitive response (HR) and induce SAR against pathogen attacks [23,24]. The on-site oxidative burst and localized cell death are two of the typical behaviors during HR caused by gene-for-gene interaction [25]. Upregulation of *RbohD* in the host during *L. biglobosa* infection could be one of the main reasons that host became resistant, and the pathogen was inhibited and limited to the infection site. The SAR induced by *RbohD* could favor the controlling of subsequent *L. maculans* infections [24].

On the other hand, *APX6* was significantly upregulated in Westar after being inoculated with *L. biglobosa*, compared to the plants inoculated with *L. maculans*. *APX6* plays a leading role in eliminating intracellular ROS and protecting plants from oxidative burst and damage consequences. As discussed above, *RbohD*, the source of ROS, was also upregulated in *L. biglobosa* inoculated cotyledons and then led to cell death. Cell death could benefit the necrotrophic pathogen *L. biglobosa* [26]. Spoel et al. (2007) described that sacrificing infected cells by triggering cell death is a defense strategy against biotrophic pathogens. In contrast, the maintenance of plant cell viability can be used as a defense mechanism against necrotrophic pathogens [26]. Based on these observations, the higher expression of *APX6* could be used to maintain the balance of cell death caused by upregulated *RbohD* in *L. biglobosa* infected cotyledons.

The hypothesis was further supported by the expression patterns of the selected defense-related genes. In the canola blackleg pathosystem, the host becomes resistant by the early recognition of *L. maculans* by gene-for-gene interaction and the interaction is known as incompatible. SA signaling pathway-related *WRKY70* initiates the process and is upregulated to higher levels as early as 3 dpi when the interaction is incompatible. However, the Westar plant’s compatible interaction with *L. maculans* in this study exhibited the upregulation of *WRKY70* from 7 dpi onwards due to the late recognition of the pathogen [19,27]. Interestingly, Westar did not show an apparent upregulation of *WRKY70* in the interaction with *L. biglobosa* at 7 dpi compared to its level at 3 dpi (data have shown only for 7 dpi). Similar results were obtained by Lowe et al., (2014). On the other hand, *WRKY33*, a gene related to the JA signaling pathway, was highly upregulated in Westar infected by *L. biglobosa* than by *L. maculans*. Previous studies revealed that responses against biotrophic pathogens are generally regulated by SA [28], while reactions towards necrotrophs are mediated by JA and ethylene [29]. Therefore, the upregulation of only *WRKY33* in response to *L. biglobosa* infections further confirms *L. biglobosa* as a necrotrophy, but *L*. *maculans* is a hemibiotroph, which initiates its lifespan as a biotroph. *PDF1.2* is considered as an ethylene-response-factor marker gene and JA marker gene [18]. The increment of *PDF1.2* in LmGFP inoculated seedlings at 7 dpi onwards can be explained by the switching of the pathogen’s trophic nature into necrotrophy [18].

*PR1* expression was upregulated highly in *L. biglobosa* inoculated Westar cotyledons compared to *L. maculans* inoculated ones. The *PR1* gene is often used as a marker for SAR. The upregulation of *PR1* and the resulting SAR will induce a defense response against subsequent infection in host plants [30]. The defense-related genes of *RbohD*, *APX6*, *PR1*, *WRKY33*, *PDF1.2*, and *CHI* began to upregulate from 3 dpi onwards (results not shown except for *RbohD*), which is consistent with the observed reduction in the *L. maculans* quantity from 3 dpi onwards in planta.

Chitinase showed a significant upregulation at 7 dpi in plants inoculated by *L. biglobosa* compared to the other plants. Chitinase belongs to the glycosyl hydrolase family, catalyzing the hydrolysis of glycosidic bonds in chitin, a key component of the fungal cell wall [31]. Lowe et al. (2014) observed a significantly higher chitinase expression in *L. biglobosa* ‘canadensis’ inoculated plants compared to the ones inoculated by *L. maculans*. Therefore, the upregulation of chitinase must play a significant role in plant resistance against *L. biglobosa* in Westar.

As demonstrated by Zou et al. (2019), different *L. biglobosa* isolates showed different levels of virulence on Westar. Interestingly, the population of *L. biglobosa* ‘brassicae’ reduced from 1 dpi onwards in susceptible Westar cotyledons in this study. In contrast, *L. maculans* showed its general virulence when LmGFP was inoculated solely on Westar cotyledons. As the Westar makes a compatible interaction with the LmGFP, no early recognition of the pathogen by the host plant and no triggering of effector-triggered immunity [27]. Therefore, the pathogen colonized within the host tissues at its biotrophic stage without doing any apparent damage upon host cells and showed an incremental increase in the *L. maculans* quantity observed from 7 dpi onwards as demonstrated by previous studies [19,27,32]. The lesion development was prominent as the virulent pathogen kills more and more tissues needed for the survival of the later necrotrophic stage [33].

Interestingly, the virulence of *L. maculans* was hidden entirely when it was co-inoculated or post-inoculated one day after *L. biglobosa*. The restraining of the *L. maculans* in the cotyledons was evidenced by restricted lesion development images and PCR quantification studies. The inhibition of *L. maculans* did not occur when the cotyledons were inoculated with *L. maculans* prior to *L. biglobosa* inoculation. The above observation suggests that *L. biglobosa* inoculation induces the host defense system and prime for any subsequent infections by *L. maculans*.

Both *L. maculans* and *L. biglobosa* coexist in most regions in which Brassica crops are grown. For instance, Dilmaghani et al. [34], revealed the existence of *L. maculans* and *L. biglobosa* species within the same field in the American continent, extending from Chile to Canada. *L. maculans* is considered more damaging to the crop than *L. biglobosa* [35]. Though damage, symptoms, and other biological features of *L. maculans* and *L. biglobosa* have been studied thoroughly; different ecological niches and other epidemiological features such as the timing of infection remained unrevealed [36]. As West et al. [37], and Mazáková et al. [38], demonstrated two *Leptosphaeria* spp. exhibited different niches even in the same host plant. Liu et al. [39], demonstrated the possibility of controlling the incidence of stem canker caused by *L. maculans* with the antagonistic interactions or the induction of systemic resistance caused by *L. biglobosa* in the host plant. Furthermore, Jacques et al. [36], suggested that though there is no sign of exclusion between two *Leptosphaeria* spp., there could be defense response induction, resource competition, and topographic exclusion. Martyn [40] and Mahuku et al. [11], reported that closely related pathogens could better induce resistance to the target pathogen.

The reduction of in planta mycelial development and disease lesion development, pathogen quantification, as well as transcriptomic observations of plant defense gene expressions confirms *L. biglobosa* could be used as a biological control in the diversification of the integrated management system of *L. maculans* in order to reduce the selection pressure exerted on host resistant genes. Further studies are required to determine the time of application of *L. biglobosa* in the field to prime the plant against this devastating pathogen.

## 4. Materials and Methods

### 4.1. Pathogen Isolates

Green fluorescent protein (GFP)-tagged *L. maculans* ‘brassicae’ isolate (refers to LmGFP) and red fluorescent protein, DsRed, tagged *L. biglobosa* ‘brassicae’ (refers to LbDsRed), received from the Dr. Kim Hammond-Kosack group [41] were used in this study. The isolates were grown in V8 agar plates and maintained under a light bank for a 24-h period at 20 ± 2 °C. After 21 days, the mycelial cultures with pycnidia were harvested by flooding culture plates with sterilized distilled water and scraping the fungal cultures gently by a heat sterilized spatula. Then, two layers of Miracloth-filtered pycnidiospore suspension was adjusted to a final concentration of 2 × 10^7^ pycnidiospores/mL using a hemocytometer.

### 4.2. Plant Growth and Inoculation

**Figure 6:**
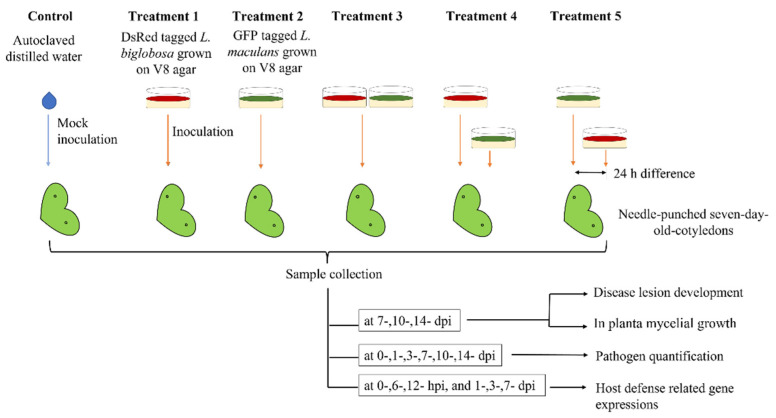
Schematic representation of the research design. Seven-day-old Westar cotyledons were wound inoculated with Control: distilled water treatment; Treatment 1: LbDsRed inoculation; Treatment 2: LmGFP inoculation; Treatment 3: LbDsRed and LmGFP co-inoculation; Treatment 4: LbDsRed followed by LmGFP inoculation (in 24-h intervals); Treatment 5: LmGFP followed by LbDsRed inoculation (in 24-h intervals). Inoculated cotyledons were collected at different time points to analyze the lesion development in planta mycelial development, pathogen quantification, and host-defense-related gene expressions.

The susceptible *B. napus* cultivar, Westar, with no known *R* genes or quantitative resistance [42] was grown in Sunshine mix #4 (Sun Gro Horticulture, BD Canada Ltd., Vancouver, BC, Canada), under a 16-h photoperiod (18 °C dark and 21 °C light) in a growth chamber at the Department of Plant Science, University of Manitoba. The cotyledons of seven-day-old seedlings were needle-punctured as one per lobe and inoculated with 10 µL of pycnidia suspension of each isolate or sterile distilled water as the control, respectively. Five different inocula types were used in addition to the control (control): treatment 1: *L. biglobosa* inoculation, treatment 2: *L. maculans* inoculation, treatment 3: *L. maculans* and *L. biglobosa* co-inoculation, treatment 4: *L. biglobosa* inoculation followed by *L. maculans* inoculation with a one-day interval, and treatment 5: *L. maculans* inoculation followed by *L. biglobosa* inoculation with a one-day interval. In treatment 3, *L. maculans* and *L. biglobosa* spore suspensions were mixed in equal volumes to prepare the co-inoculum (Figure 6). For treatments 3 and 4, the first inoculation was followed by another pinching of the same site to perform the second inoculation as 24 h of callus formation of the pinched site acts as a barrier for the second inoculation [43].

### 4.3. Disease Lesion Development on Cotyledons

Cotyledon lesion developments in each treatment were observed at 7-, 10-, and 14- days post inoculation (dpi) and the lesion development was calculated as the percentage of the total cotyledon area by ImageJ software [44].

### 4.4. Confocal Observation of in Planta Mycelial Development

The collected cotyledons from each treatment at 7-, 10- and 14- dpi were observed by laser confocal LSM 700 microscope (LSM 700; Zeiss, Jena, Germany). At 7 dpi, cotyledon pieces 2 *×* 2 mm^2^ in size were cut two millimeters away from the pinched wounds made on the cotyledons. At 10- and 14- dpi, the cut pieces of cotyledons with lesions were taken two millimeters away from the margin of the diseased lesions with the growing fungus. The cut pieces were water-mounted on slides. The cut pieces were water-mounted on slides, and GFP-tagged *L. maculans* mycelia and DsRed-tagged *L. biglobosa* development were observed by Zeiss LSM 700 confocal microscope (Jena, Germany). LmGFP was imaged using the excitation of BP 470/40 nm and emission of BP 525/50, and LbDsRed was visualized with the excitation of BP 550/25 nm and emission of BP 605/70. The study was repeated two times.

### 4.5. PCR Identification of Leptosphaeria maculans ‘brassicae’ and L. biglobosa ‘brassicae’ Isolates in Inoculated Cotyledons

Cotyledons of LmGFP and LbDsRed separately and co-inoculated were collected at 7 dpi as two cotyledons per replicate for three replicates. Genomic DNA extraction from inoculated Westar cotyledons was performed using the CTAB method that Liban et al. [45], explained with modifications. Freeze-dried cotyledons were grounded into a fine powder and mixed with lysis buffer (Tris, EDTA, CTAB, and NaCl), incubated at 65 °C for 20 min, extracted with phenol: chloroform: isoamyl alcohol (25:24:1), precipitated with 5M NaCl and 95% ethanol, and washed by 70% ethanol twice. After the final centrifugation, the DNA pellet was dissolved in 100 μL sterile distilled water.

Species-specific primers LmacF/LmacR ((LmacF, 5′-CTT GCC CAC CAA TTG GAT CCC CTA-3′; LmacR, 5′-GCA AAA TGT GCT GCG CTC CAGG-3′ for *L. maculans*) and LbigF/LmacR (LbigF, 5′-ATC AGG GGA TTG GTG TCA GCA GTT GA-3′; LmacR, 5′-GCA AAA TGT GCT GCG CTC CAGG-3′ for *L. biglobosa*) were used for PCR identification of the isolates as described by Liu et al. (2006). PCRs were performed in 20 μL volumes, made up of 10 μL of 2x Taq Mix (FroggaBio, Toronto, ON, Canada), with 0.8 μL of each primer (10 mM) and 100 ng total of sample gDNA and sterile distilled water to increase the volume. PCR was performed on a T100 Thermal cycler (Bio-Rad, Hercules, CA, USA), and the amplification program set was an initial denaturation period of 95 °C for two minutes followed by 30 cycles of 95 °C for 15 s, 70 °C for 30 s, and 72 °C for one minute, followed by an additional primer-extension period of 72 °C for 10 min. PCR products were resolved by electrophoresis through a 1% (*w*/*v*) agarose gel (Figure 2).

### 4.6. Quantification of Pathogen Isolates Using Quantitative PCR

Fungal suspensions of both isolates of LmGFP and LbDsRed with mycelia, pycnidia, and pycnidiospores were collected from a single spore grown on a V8 agar plate of each isolate in sterile distilled water. Genomic DNA of fungal suspensions was extracted using the modified CTAB method, as Liban et al. [45], described, with minor modifications. The extraction procedure followed was the same as explained above, except for the initial steps. The fungal suspensions were centrifuged to remove the aqueous portion of the fungal biomass. The samples were mixed with a lysis buffer and lysed with between 5 and 8 ceramic beads at 10,000 rpm for one min. The rest of the procedure was the same as described above.

Cotyledons of each treatment were collected at 0-, 1-, 3-, 7-, 11- dpi as two cotyledons per replicate for three replicates. Genomic DNA extraction was extracted from inoculated Westar cotyledons as described above. The amounts of DNA of *L. maculans* and *L. biglobosa* present in each pure fungal pathogen sample and pathogen inoculated cotyledon samples were quantified using SYBR Green qPCR, with species-specific primer pairs LmacF/LmacR and LbigF/LmacR used for *L. maculans* and *L. biglobosa*, respectively, as described by Liu et al. (2006). Quantitative PCR reaction mixtures were prepared to a total volume of 20 μL made up of 10 μL of Bio-Rad CFX ConnectTM Real-Time System with SYBR^®^ Green Supermix (Bio-Rad, Hercules, CA, USA), 0.8 μL (10 mM) of forward primer, and 0.8 μL (10 mM) of reverse primer, 100 ng total of sample gDNA, and sterile distilled water to increase the volume as per manufacturer’s instructions.

PCRs were performed with the amplification programs set as follows: 50 °C for two minutes, 95 °C for three minutes, 40 cycles at 95 °C for five seconds, 60 °C for 10 s, and 72 °C for 30 s. Melting curve analysis was performed by increasing 0.5 °C at 3 s/step from 55 to 95 °C. In each qPCR run, a standard dilution series consisting of 10,000, 1000, 100, 10, and 1 pg of DNA of *L. maculans* “brassicae” or *L. biglobosa* “brassicae” pure culture was used to produce a standard curve. The amount of *L. maculans* DNA or *L. biglobosa* DNA for each inoculated cotyledon sample was estimated using the standard curve. Results were expressed as the amount (pg) of *L. maculans* or *L. biglobosa* DNA in 100 ng total DNA from diseased plant tissue. The study was repeated two times [46].

### 4.7. Defense-Related Gene Expression

Inoculated cotyledons collected at 0-, 6-, 12- h post-inoculation (hpi) and 1-, 3-, 7- dpi were promptly frozen in liquid nitrogen. Frozen cotyledons were homogenized in liquid nitrogen with a sterilized porcelain mortar and pestle. Plant total RNA was extracted with PureLink^®^ Plant RNA Reagent (Invitrogen, Carlsbad, CA, USA) and treated with a TURBO DNA-freeTM Kit (Ambion, Austin, TX, USA) according to the manufacturer’s instructions. Reverse transcription of the first-strand cDNA was achieved using the first cDNA Synthesis Kit (Thermo Scientific, Waltham, MA, USA) with one μg total RNA. For the 10 μL of total qPCR volume, 2.1 μL of 100-fold diluted cDNA, five μL of Bio-Rad CFX Connect^TM^ Real-Time System with SYBR^®^ Green Supermix (Bio-Rad, Hercules, CA, USA), and 0.4 μL of each primer (10 mM; defense-related genes and used primers shown in Table 1) were used for PCR reaction. Real-time quantitative PCR was performed on a CFX96 Real-Time Instrument (Bio-Rad, USA) with the amplification program of 95 °C for two minutes, 40 cycles of 95 °C for five seconds, 60 °C for 10 s, and 72 °C for 30 s. Melting curve analysis was performed by increasing 0.5 °C at 3 s/step from 55 to 95 °C. (The primers list should be mentioned (Table 1)). The relative gene expression level was calculated using the 2^−ΔΔCT^ method [47]. The study was repeated two times.

### 4.8. Statistical Analysis

Unless specified, the analyses of samples used three biological replicates. Statistical analyses were performed using SAS 9.0 software. The data were log-transformed to maintain the homogeneity and normality of residuals. Analysis of variance (ANOVA) was conducted using the MIXED model to determine the significance of the treatments. The Fisher’s least significant difference (LSD) at the 0.05 probability level was applied to the percentage of lesion development, defense gene expression, and DNA quantity of each isolate.

## 5. Conclusions

Fluorescent protein-tagged *Leptosphaeria* isolates demonstrated a successful in planta control of virulent *L. maculans* in cotyledons pre- or co-inoculated with *L. biglobosa.* Moreover, lesion development on cotyledons, host defense-related gene expressions, and pathogen quantification studies in time course studies confirmed the above results. The outcome of the study concludes that *L. biglobosa* ‘brassicae’ can inhibit the destructive pathogen *L. maculans* in susceptible canola varieties. The reliability of this biological control agent under field conditions and the applicability of *L. biglobosa* into the integrated management approaches have to be studied further.

## Figures and Tables

**Table 1:** List of selected defense-related genes in canola used in gene expression analyses and the primer pairs used for each gene.

Gene	Full Name	Defense Signaling Pathway	Forward Primer (5′3′)	Reverse Primer (5′3′)
*WRKY70*	WRKY transcription factor 70	Salicyclic acid signaling pathway	ACATACATAGGAAACCACACG	ACTTGGACTATCTTCAGAATGC
*PR1*	Pathogenesis-related protein 1	Salicyclic acid pathway	GGCTAACTATAACCACGATTC	GTTCCACCATTGTTACACC
*WRKY33*	WRKY transcription factor 33	Jasmonic acid signaling pathway	TGTCGGACAGCTTGGGAAAG	AGAGGACGGTTACAACTGGAGAAA
*PDF1.2*	Plant defensin 1.2	Ethylene and Jasmonic acid pathway	AAATGCTTCCTGCGACAACG	AGTCCACGTCTCCGATCTCT
*RbohD*	Respiratory burst oxidase homolog protein D	Reactive Oxygen Species Production	TATCCTCAAGGACATCATCAG	TATCCTCAAGGACATCATCAG
*APX6*	Ascorbate peroxidase	Reactive Oxygen Species Scavenging	AGTTCGTAGCTGCTAAATATT	GGAGTTGTTATTACCAAGAAA
*CHI*	Chitinase	Pathogen chitin degradation	TGCTACATAGAAGAAATAAACGG	TTCCATGATAGTTGAATCGG
*Actin*	Actin	Reference gene used in the assay	CTGGAATTGCTGACCGTATGAG	GTTGGAAAGTGCTGAGGGATG

## Data Availability

Not applicable.
